# A spectrophotometric method for determining residual protein levels on reusable surgical instruments: a comparison of six washing methods of instruments with an internal lumen

**DOI:** 10.3205/dgkh000587

**Published:** 2025-09-30

**Authors:** Deborah Montmeat, Clément Boisseillier, Nabil Benhajkassen, Jimmy Rose, Guillaume Pariscoat, Jennifer Le Grand, Cyril Cambier

**Affiliations:** 1 Pharmacy Department, Bichat-Claude Bernard University Hospital, Assistance Publique Hôpitaux de Paris, Paris, France; 2 Paris-Saclay University, Paris, France; 3 Radiopharmacy Unit, Bichat-Claude Bernard University Hospital, Assistance Publique Hôpitaux de Paris, Paris, France

**Keywords:** surgical instrument, sterilization, instrument cleaning, reamers, spectrophotometric method, protein test, soiling test, quantitative determination

## Abstract

**Aim:**

Washing is a multistep process and a critical step in the sterilization of reusable instruments used in surgery. The complexity of the design of some types of instruments, such as those with a lumen, is a major challenge, both in terms of cleaning and of checking their cleanliness.

The aim of this study was to validate a new approach to compare washing methods of reusable instruments with internal lumen. This approach was then tested to compare six methods for washing reamers.

**Methods:**

The proposed method was based on detection by Soiltest^®^, a commercially available soiling test, using ninhydrin in a spectrophotometric approach at 570 nm. To increase the sampling yield of conventional swabbing, we used new wash brushes for each sampling.

**Results:**

Soiltest^®^ results were linearly quantified with a quantitative and reproducible method. The equation of calibration curve was Y=0.2043X–0.03489, the coefficient of determination was 0.9926, and the slope was significantly different from zero (p<0.0001, F-test). The optimal cleaning method for reamers was a combination of chemical detergents, manual cleaning ensuring that no obstruction is present in the instrument, and mechanical washing in a washer-disinfector with a system of continuous irrigation of the lumen of the instrument.

**Conclusion:**

This study validated a new approach to evaluate the cleanliness of critical reusable surgical instruments with lumen based on the detection of protein residues using Soiltest^®^, by means of a colorimetric method using ninhydrin.

## Zusammenfassung

**Zielsetzung:**

Das Waschen ist ein mehrstufiger Prozess und ein kritischer Schritt bei der Sterilisation wiederverwendbarer Instrumente, die in der Chirurgie eingesetzt werden. Die Komplexität des Designs einiger Arten von Instrumenten, z. B. derjenigen mit Lumen, stellt eine große Herausforderung dar, sowohl was die Reinigung als auch was die Überprüfung ihrer Sauberkeit betrifft.

Ziel der Studie war die Validierung einer neuen Methode zum Vergleich der Reinigungsverfahren für wiederverwendbare Instrumente mit Innenlumen. Diese Methode wurde getestet, um sechs Reinigungsmethoden für Hohlfräser zu vergleichen.

**Methode:**

Die vorgeschlagene Methode basiert auf dem Nachweis mittels Soiltest^®^, einem handelsüblichen Test mit standardisierter Anschmutzung und spektrophotometrischem Nachweis von Ninhydrin bei 570 nm. Um die Ausbeute der Probenahme bei herkömmlichen Abstrichen zu erhöhen, wurde für jede Probenahme eine neue Reinigungsbürste verwendet.

**Ergebnisse:**

Soiltest^®^ wurde mit einer quantitativen, reproduzierbaren Methode linear quantifiziert; die Gleichung der Kalibrierungskurve lautete Y=0,2043X–0,03489; das Bestimmtheitsmaß betrug 0,9926; die Steigung war signifikant von Null verschieden (p<0,0001, F-Test). Die optimale Reinigungsmethode für Hohlfräser war eine kombinierte Methode mittels manueller Reinigung mit Detergentien, die sicherstellt, dass keine Verstopfung im Instrument vorhanden ist, und mechanische Reinigung in einem Reinigungs-Desinfektions-Gerät mit einem System zur kontinuierlichen Spülung des Instrumentenlumens.

## Introduction

Decontamination and washing are multistep processes aimed at removing organic matter, and are critical steps in the sterilization of reusable instruments used in surgery [1]. Protein deposits promote bacterial adhesion [2] and jeopardize the final sterilization. Hospital-acquired infections (HAI) are a major challenge in the 21^st^ century. While they represent a burden in terms of economic cost [3], the epidemiological data are frightening. They are the sixth leading cause of death in the United States [3]; there is an incidence of 250 per 100,000 acute occupied bed-days in a study of hospitals in the UK [4]; and there are 750,000 infections per year in France [5].

A recent review highlighted the inconsistencies in reprocessing protocols, and the lack of standardization of testing, particularly with respect to the risk of prion transmission via surgical equipment [6], [7]. The complexity of the design of some types of instruments, such as those with a lumen, is a major challenge in terms of cleaning, reprocessing, and checking their cleanliness. Many resources are available for Central Sterile Services Departments (CSSD) to ensure proper cleaning, but the washing steps associated with particular instruments are not standardized [8], [9]; thus, disparities exist in current practice. Although recommendations on washing assessment differ between countries, most often the recommendation is to carry out a daily visual check of instruments and to add additional tests. With this in mind, stain tests have appeared on the market, designed to mimic blood that may remain after washing stages. These substances are highly colored and allow visual assessment of an instrument’s cleanliness, but they do not function with instruments with lumina, such as reamers. Studies using microscopy have questioned this inspection method [10], with one, for example, showing that when an instrument is recorded as “clean”, it actually contains residual organic debris in 84% of cases [11].

Numerous tests for assessing instrument cleanliness are now available on the market. They are often based on the detection of residual proteins on instruments. Ninhydrin tests are specified in EN ISO 15883-5:2021 [12] and Health Technical Memoranda 0101/2030; however, they have been shown to be inadequate and inappropriate for routine use [13]. Many studies have been published on ninhydrin-based tests [14], and a number of limitations have emerged. For example, the study by Lipscomb et al. [13] pointed to a lack of sensitivity linked in particular to the way in which instruments are sampled. Ninhydrin is a chemical compound that reacts with the N-terminal part of a protein to form a visible violet coloration. Intrinsic to the chemical reaction mechanism and described in previous articles [14], it appears that its sensitivity varies according to the molecular weight of the protein studied. Blood residues can contain any kind of protein, and it is difficult to talk about sensitivity in terms of µg/cm² of sample. Moreover, commercially available tests are based on visual assessment of ninhydrin staining, but the colored product absorbs at 570 nm, so that the human eye would not be able to detect low protein concentrations. Finally, several authors have demonstrated a reduced sensitivity associated with the swab method of sample collection [14], [15].

**Figure 1:**
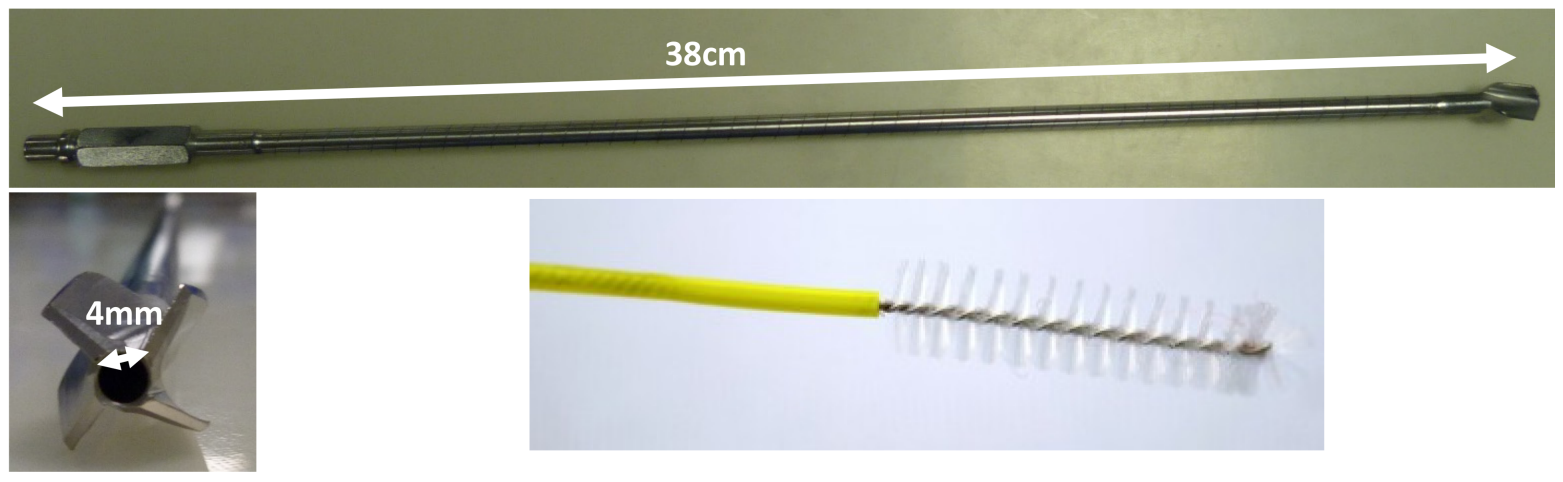
Photos of hollow reamers used for the study and wash brushes used to clean hollow reamers

In this study, we propose a new method based on detection by a soiling test (Soiltest^®^; Browne^©^ factory) using ninhydrin to compare different washing methods. This study was carried out on reamers (Figure 1 (Fig. 1)) and attempted to increase the sensitivity of tests already described in two ways. First, samples are picked up with new wash brushes (ScopeClean^®^; Albyn Medical, Cordovilla Navarra, Spain, Figure 1 (Fig. 1)), which are then immersed directly in a ninhydrin solution. The goal was to increase the sampling yield compared with conventional swabbing, since the original purpose of this tool is to remove proteins in order to clean the instrument. Secondly, for better detection of smaller amounts, results were determined by spectrophotometry rather than with the naked eye. All results were expressed as a relative Soiltest^®^ volume, and we have chosen to use a reagent as close as possible to human blood, to maximize similarity to real-life conditions. 

## Methods

### Protein detection method

The protein detection method was based on a chemical reaction involving the ninhydrin reagent, purchased from Sigma^®^ (La Chapelle-sur-Erdre, France). Calibration ranges were determined with increasing concentrations of Soiltest^®^ (Browne, STERIS Solutions limited, Leicester, United Kingdom). The company does not reveal the exact composition of its reagent, but assures that the composition is equivalent to the Edinburgh soil described in ISO/TS 15883-5; it is a dried soil formula containing proteins, lipids and polysaccharides. For calibration, six volumes were dissolved in 2 ml of 5g/L ninhydrin solution: 0.31 µL/mL, 0.62 µL/mL, 1.25 µL/mL, 2.50 µL/mL, 5.00 µL/mL, and 7.50 µL/mL. Ninhydrin was dissolved in 65% ethanol, prepared by mixing absolute ethanol (VWR^©^, Radnor, PA, USA) with purified water (Aguettant^©^, Lyon, France). Spectrophotometric measurements were performed with a spectrophotometer (SAFAS UVmc^®^; Monaco), at a maximum absorbance of 570 nm after incubation at 60°C for 75±15 minutes. 

Calibration ranges were repeated 5 times, all points were performed in duplicate, and a mean resultant absorbance was obtained. The Limit of Detection (LOD) was calculated as the mean of the ten blank values plus 3 standard deviations. The Limit of Quantification (LOQ) was calculated as the mean of the ten blank values plus 10 standard deviations.

### Methods of washing studied for instruments with internal lumina

The instruments with internal lumens that were chosen were reamers with a of length of 38 cm and an internal diameter of 4 mm. The instruments were soiled by injecting 10 mL of Soiltest^®^ through the lumen with a syringe and left to dry overnight. Six washing methods were compared. All methods were performed on 5 different instruments and 3 samplings of 30 cm² were performed for each instrument in the lumen using a new wash brush. 

#### Method 1

Instruments were rinsed by injecting the Surfanios premium^®^(Anios, Lezenne, France) solution, a detergent-disinfectant used diluted at 0.25%, 5 times with a syringe and then placed on a washing module dedicated to instruments without internal light (DS 1000-P-001). Subsequently, they underwent an automated washing process in an instrument washer-disinfector (DS 1000 Steelcor^®^, Riese Pio, Italia), which undergoes annual certification. The washing cycle consists of a pre-wash phase at 38°C for 2 minutes, and a washing phase at 55°C for 5 minutes with the injection of an enzymatic alkaline detergent (neodisher^®^ Mediclean forte, Villepinte, France) at a concentration of 0.4%. These steps were then followed by a rinsing phase at 40°C for 2 minutes, and then a thermal disinfection phase at 90°C, during which a rinsing liquid (neodisher^®^ MediKlar, Villepinte, France) was injected at a concentration of 0.05%. Finally, this cycle concluded with a drying phase using air heated to 120°C for 25 minutes.

#### Method 2

Instruments were rinsed by injecting the Surfanios premium^®^ solution 5 times with a syringe and were then placed on a module specifically for coelioscopy instruments (DS 1000-P-009 Steelco^®^). The same washing process as in method 1 was then carried out.

#### Method 3

Instruments were soaked for 20 minutes in an ultrasonic tank filled with Surfanios premium^®^ solution, without a hollow-body irrigation system, and rinsed by injecting the Surfanios premium^®^ solution 5 times with a syringe and then placed on a washing module dedicated to instruments without internal light (DS 1000-P-001). The same washing process as in method 1 was then carried out. 

#### Method 4

Instruments were rinsed by injecting the Surfanios premium^®^ solution 5 times with a syringe and washed with a Jetwash^®^ (high-pressure steam cleaner from Anios Laboratories©) and then placed on a washing module dedicated to instruments without internal light (DS 1000-P-001). The same washing process as in method 1 was then carried out. 

#### Method 5

Instruments were manually swabbed 5 times with Surfanios Premium solution^®^, swabbing in on one side and out on the other, rinsed by injecting the Surfanios premium^®^ solution 5 times with a syringe and placed on a washing module dedicated to instruments without internal light (DS 1000-P-001). The same washing process as in method 1 was then carried out. 

### Method 6

Instruments were manually swabbed 5 times, swabbing in on one side and out on the other, rinsed by injecting the Surfanios premium^®^ solution 5 times with a syringe and then placed on a module specific for coelioscopy instruments (DS 1000-P-009 Steelco^®^). The same washing process as in method 1 was then carried out.

All washing methods are briefly presented in Table 1 (Tab. 1). 

Once dry, instruments were swabbed 3 times with 3 different wash brushes (ScopeClean^®^, Albyn medical; Figure 1 (Fig. 1)) which were placed in 5g/L ninhydrin solution and incubated 75±15minutes at 60°C. Spectrophotometric measures were then performed with a spectrophotometer SAFAS UVmc^®^ at a maximal absorbance of 570 nm. Negative controls consisted of swabbed but unsoiled instruments. Positive controls consisted of swabbed but uncleaned instruments that had been soiled by the same protocol. 

### Statistical analysis

Analyses of calibration ranges consisted of an R-squared determination and an F-test to evaluate if the slope was significantly non-zero. Analyses were conducted using Graphpad Prism (Dotmatics^®^, Boston, MA, USA).

A Kruskal-Wallis test was used to compare the Soiltest^®^ volumes measured with the different washing methods. Dunn’s test was performed in cases of significant differences between groups identified by the Kruskal-Wallis test, to compare each method with the negative controls. 

In parallel, we also established a “positivity threshold” which allowed a binary classification of sterilization methods into “positive” or “negative”. The limit of positivity was calculated as the mean of the negative control values plus 3 standard deviations. All volumes higher than this value were considered “positive”.

Statistical analysis was conducted using R version 3.4.4.

## Results

### Calibration ranges

**Figure 2:**
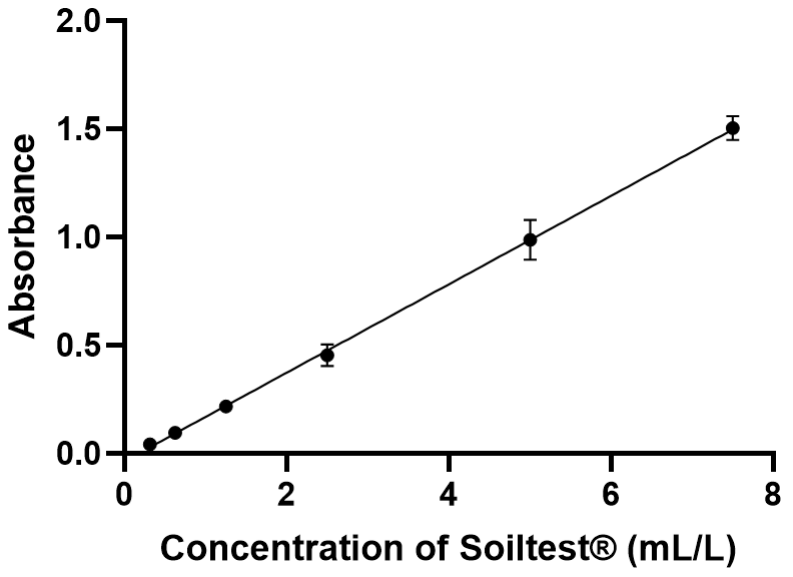
Calibration curve of Soiltest^®^’s volumes at 570 nm. Calibration ranges were repeated 5 times, all points were performed in duplicate. On the graph, all points are represented as mean ± standard deviation

The proposed method was successful in quantifying Soiltest^®^ with a linear method based on spectrophotometry at an absorbance at 570 nm (Figure 2 (Fig. 2)). The equation was Y=0.2043X–0.03489 with a coefficient of determination of 0.9926. The slope was significantly different from zero, with a p-value <0.0001 (F-test). LoD was 0.022 and LoQ was 0.033 (Table 2 (Tab. 2)).

### Comparison of washing methods

**Figure 3:**
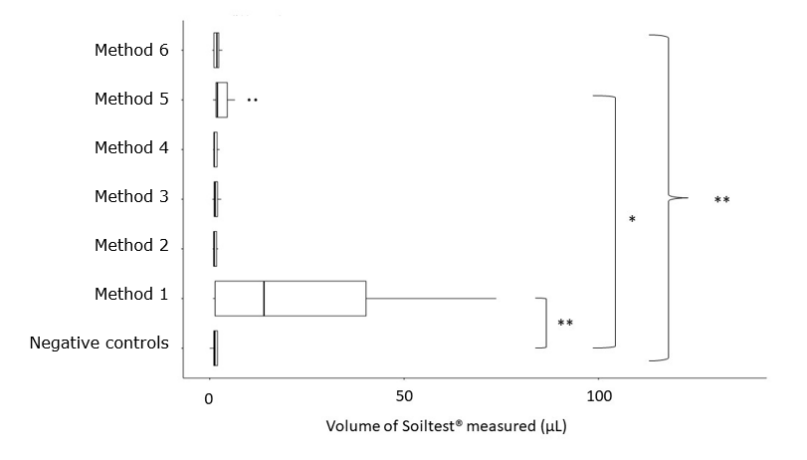
Comparison of 6 washing methods of reamers**: p<0.01, *: p<0.02. A significant difference between all groups was determined (Kruskall Wallis test, p<0.01). Method 1 and method 5 were significantly different compared to the negative controls (Dunn’s test, p<0.01, p<0.02 respectively).

A significant difference was found between all washing methods (Kruskall-Wallis test, p<0.01). Then, methods were compared one by one with the negative controls. A significant difference was shown for methods 1 and 5 compared with the negative controls (Dunn’s test, p<0.01, p<0.02 respectively) (Figure 3 (Fig. 3)). 

**Figure 4:**
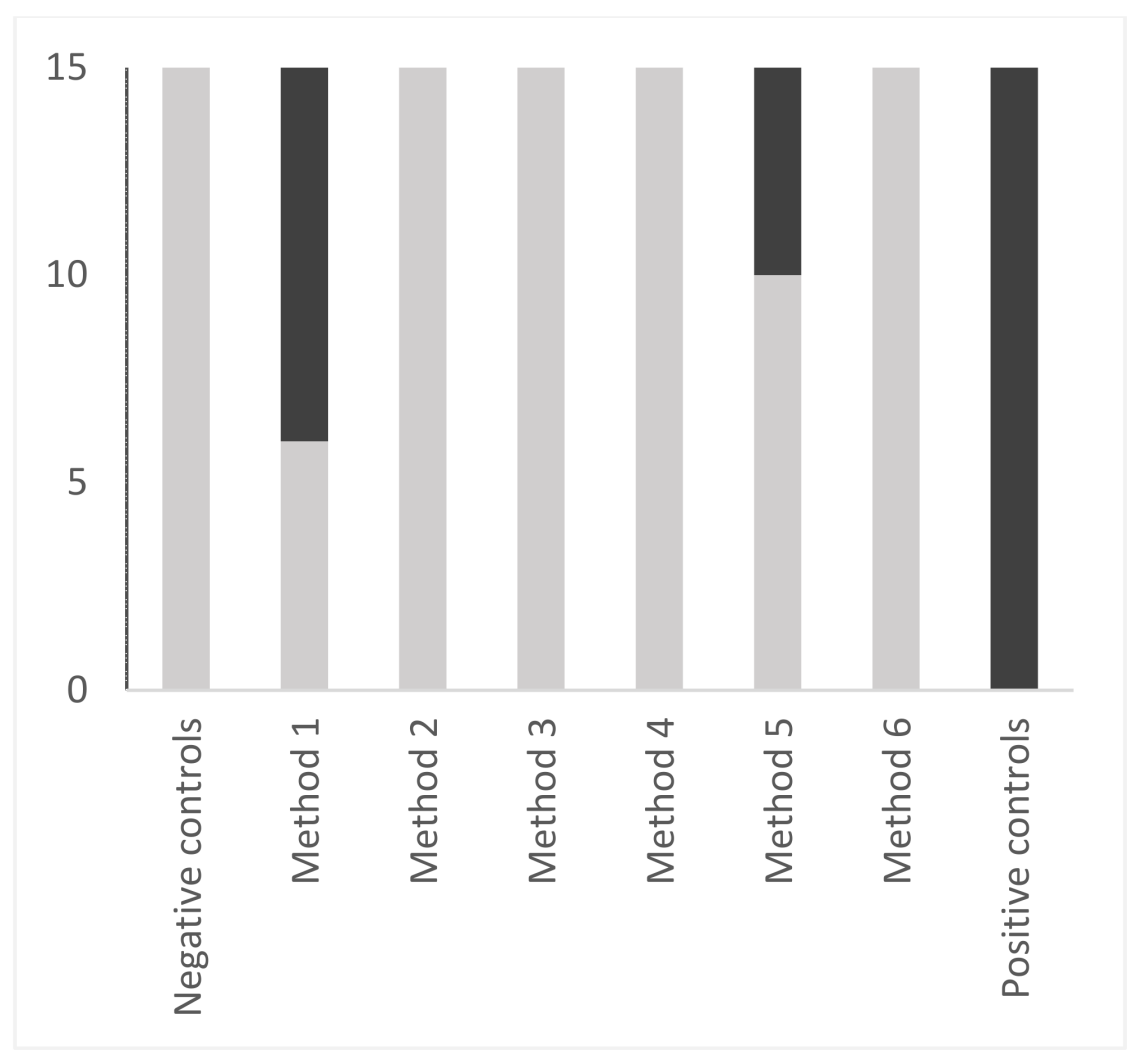
Comparison of 6 washing methods of reamers using a qualitative statistical approach. The positivity limit was determined from the mean of values of negative controls (mean+3 standard deviation). Negative samples are shown in light grey. Positive samples are shown in dark grey.

A qualitative approach was also tested, since in sterilization, all instruments must be clean to be sterilized. Nine out of 15 method-1 samples were dirty, and 5 out of 15 method-5 samples were soiled (Figure 4 (Fig. 4)). All positive controls were soiled and all negative controls were clean. 

## Discussion

HAI are a major challenge in the 21^s^t century. Among all diagnoses of HAI, a survey in 2016 in the United States showed that surgery-site infections represented 31% of admissions and were the most frequent unplanned cause of postoperative readmission. It is now clearly established that HAIs contribute to the emergence of multidrug-resistant bacteria [16], [17], [18], and daily efforts must be made to limit them. As long as hospitals are reservoirs of cross-contamination, pre-treatment of surgical instruments performed immediately prior to use and their subsequent sterilization remain key strategies for the control of HAI [19]. The control of washing processes and monitoring of cleaning standards are priorities in order to guarantee the final sterility of surgical instruments, for tissue residues including proteins are a site of bacterial adhesion [2], [11]. To achieve this, we need to strike a balance between control tests that are too insensitive and those that are too expensive or too complicated to implement in everyday practice. Visual inspection is recommended on a daily basis and is easy to set up. However, it cannot be the only control, since it is not very sensitive, particularly for residues of a colorless liquid, and is impossible to set up for some critical instruments such as those with a lumen. Nevertheless, its use remains essential, as it is easy to perform. Several studies mention microscope-based approaches [20], [21], which have proved to be very sensitive but are both expensive and difficult to implement in everyday practice. 

In this study, we have chosen to use a colorimetric approach to the detection of residual proteins, considering that these tests could be adapted for rapid implementation and would not be too expensive for our Central Sterile Services Department. Well-studied approaches include ninhydrin detection, using both commercial and non-commercial tests. In this context, the study by Lipscomb et al. [13] is of great interest, as it highlighted the lack of sensitivity of this test, but also questioned its sampling method. The article by Nayuni et al. [14] also raised questions about the differences in sensitivity observed according to the proteins used for analysis. To be reflect real life as closely as possible, a reagent specially marketed for sterilization department controls was chosen, namely, Soiltest^®^. Its manufacturer does not reveal the exact composition of its reagent, but assures that the composition is equivalent to the Edinburgh soil described in ISO/TS 15883-5. 

The present study developed a quantitative and reproducible method using spectrophotometry to improve visual perception of colored residues. As spectrophotometers are easily accessible in hospitals, this method should be easy to perform in several facilities. It was then tested for an everyday problem: which washing method is optimal for instruments with a lumen? The methods for cleaning reamers were chosen, because they are particularly difficult to clean due to their design (38 cm with an internal diameter of 4 mm) and because many cleaning methods are available. Considering the lack of sensitivity associated with instrument sampling, we used single-use wash brushes to detach residues from the inner walls of an instrument with a lumen. Since wash brushes are not sterile, negative controls were performed by swabbing clean instruments, and positivity thresholds were determined from their absorbance. The results were not unexpected and were in line with previous studies. Instruments cleaned with steam cleaners (Jetwash^®^) were found to contain significant protein residues, and the hypothesis was that heat could fix proteins to the walls. Instruments only rinsed with a syringe and placed in a basket in a washer-disinfector without continuous irrigation of the internal lumen did not have satisfactory washing results. 

## Conclusion

This study tested a new approach to evaluating the cleanliness of critical reusable instruments for surgery based on the detection of protein residues of Soiltest^®^ by means of a colorimetric method using ninhydrin. Here, the cleanliness of reamers cleaned by 6 different methods were evaluated, showing that the optimum cleaning method combined chemical detergents, manual cleaning and mechanical washing in a washer-disinfector with a system of continuous irrigation of the lumen of the instrument. However, swabbing remains an essential step, since it ensures that there is no obstruction inside the instrument.

## Notes

### Authors’ ORCIDs

- Montmeat D: https://orcid.org/0000-0003-3989-6855
- Pariscoat G: https://orcid.org/0000-0003-3152-9591
- Cambier C: https://orcid.org/0009-0004-8778-9661

### Funding

None. 

### Acknowledgments

We would like to thank Jeremie Montmeat for his expertise in statistics. No funding was received for this work.

### Competing interests

The authors declare that they have no competing interests.

## Figures and Tables

**Table 1:**
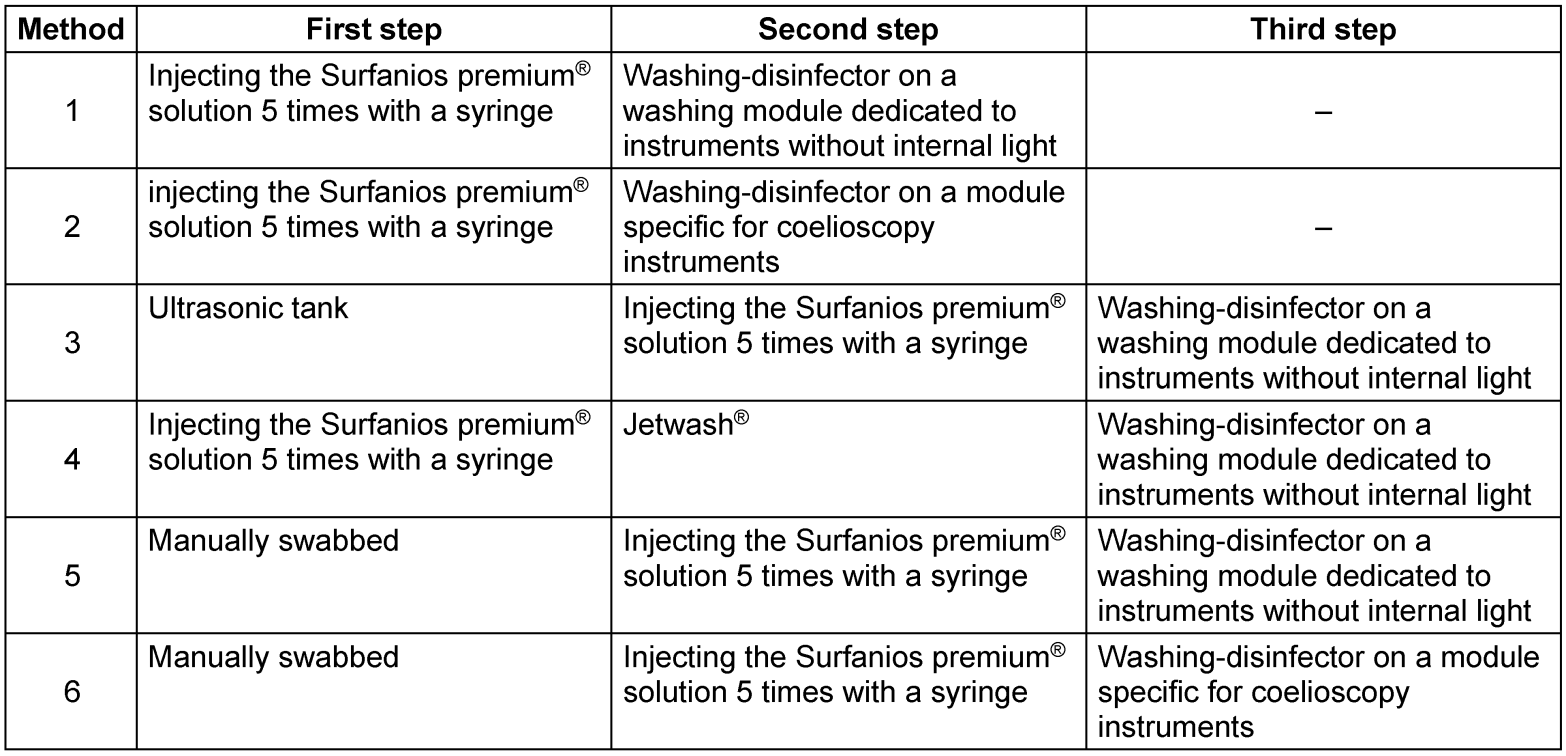
Summary description of the six methods compared

**Table 2:**
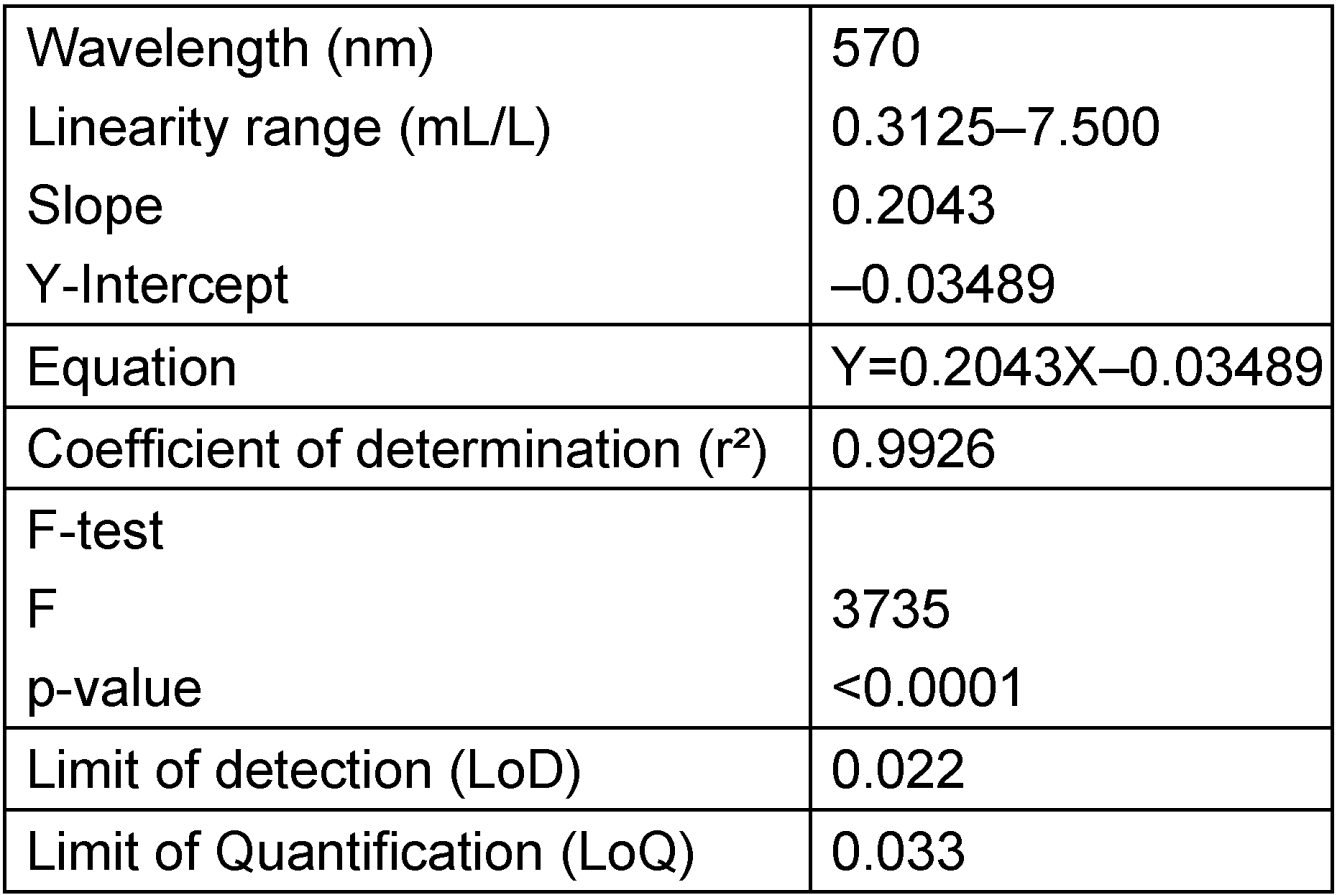
Regression and validation data for quantitative analysis of Soiltest^®^ by the proposed method
